# Supplementary material for: Cation Disorder and Local Structural Distortions in AgxBi1–xS2 Nanoparticles
Source: Nanomaterials (Basel). 2020 Feb 12;10(2):316. doi: 10.3390/nano10020316 (PMC7075158; doi:10.3390/nano10020316)
Supplement: Supplementary file 1 [file nanomaterials-10-00316-s001.pdf]

Cation disorder and local structural distortions in $Ag_xBi_{1-x}S_2$ nanoparticles

J. Kopula Kesavan et al.

Supplementary Material

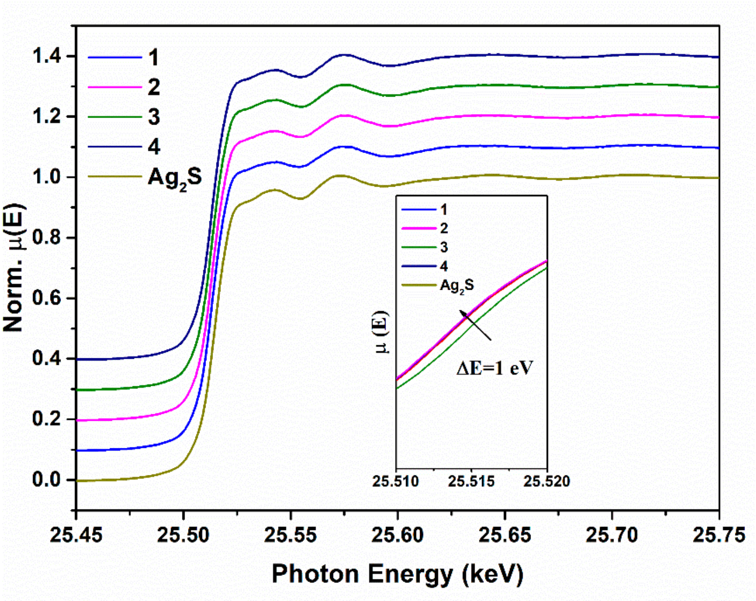

Figure S1. Ag K-edge XANES spectra of the samples.

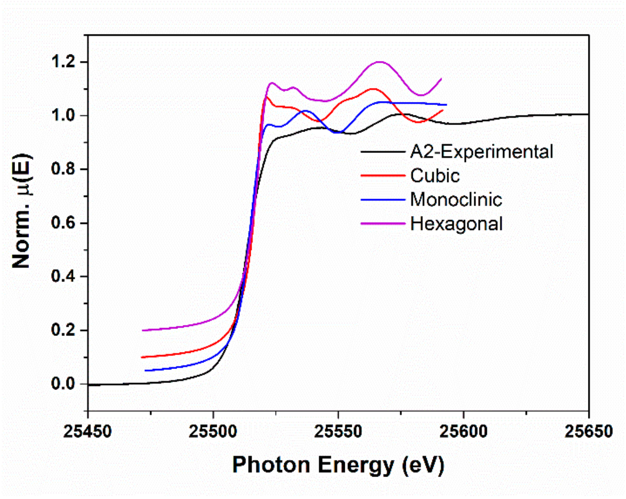

Figure S2. Comparison of the Ag K – edge XANES spectrum of sample 2 with FDMNES simulations for schapbachite (cubic), acanthite (monoclinic) and matildite (hexagonal) bulk phases.

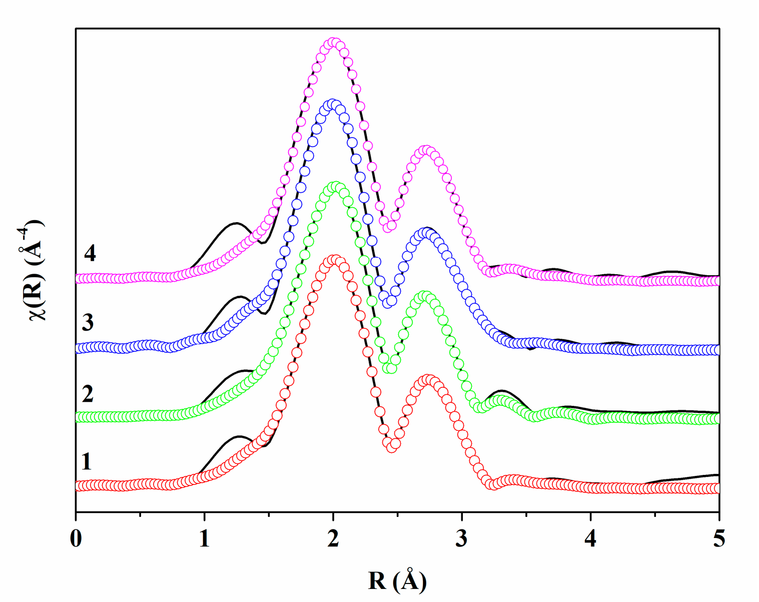

Figure S3. Best fits of the Ag K – edge EXAFS spectra for all samples.

Bi L_3 – edge XAFS

We briefly discuss Bi L_3 – edge EXAFS. Bi_2S_3 has orthorhombic structure with the spacegroup 62 i.e. Pnma. FEFF generated scattering paths for this structure have been used to fit the reference Bi_2S_3 EXAFS spectrum, reported in Fig. SM – 4. It is found that the Bi_2S_3 spectrum can be fitted using following single scattering paths: three S atoms at 2.64 Å, three S atoms at 3.03 Å, one more S atom at 3.34 Å, and 3 pairs of Bi atoms at the distances of 3.95 Å, 4.122 Å and 4.35 Å respectively. No multiple scattering paths were found to have significant intensity. The many body amplitude reduction factor was $S_0^2=0.715$.

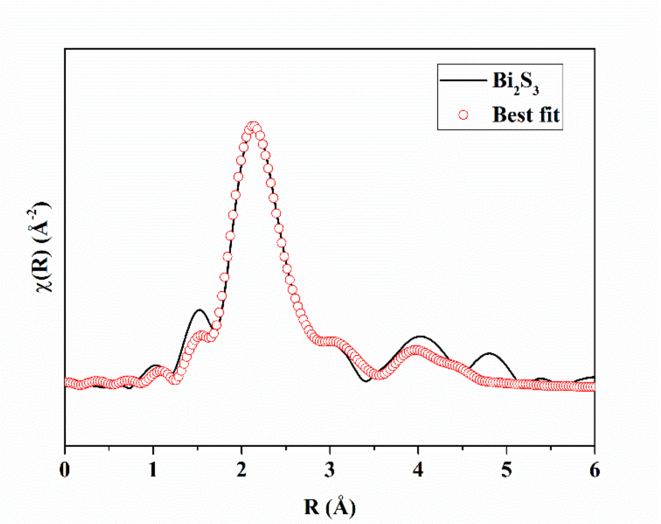

Figure S4. FT of Bi L_{III} edge EXAFS spectrum of a Bi_2S_3 reference sample with its best fit.

The same $S_0^2(0.715)$ was used to fit the Bi L_{III} EXAFS spectra of samples, using the same model (see Fig. 1 right panel) which was used to fit the Ag K-edge spectra of samples. There are four S atoms (2.50 Å – 2.97 Å) around Bi and the spectra were fitted using four single scattering paths corresponding to these distances. Fig. SM – 5 shows the Fourier Transformed Bi L_{III} edge EXAFS spectra of the samples with their best fits. The

numerical results are shown in Table S1. Due to the available quality of data the fit was limited to the first shell and thus it was not possible to obtain information on the local environment of the cation sub lattice.

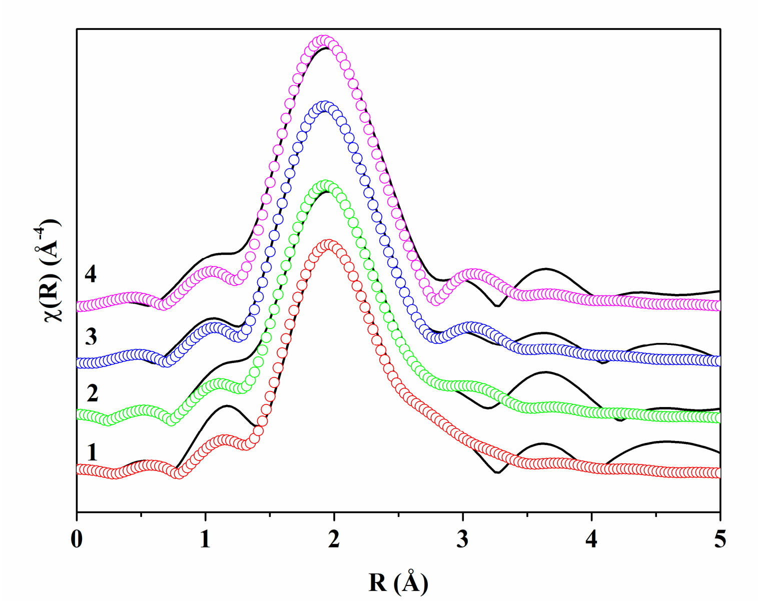

Figure S5. FT of Bi L$_{III}$ edge EXAFS spectra of the samples with their best fits.

Table S1: Summary of quantitative analysis of Bi L$_{III}$-edge EXAFS spectra of the samples. Uncertainties on the least significant figures are in brackets.

Sample	R_{Bi-S1}	σ^2_{Ag-S1}	R_{Bi-S2}	σ^2_{Bi-S2}	R_{Bi-S3}	σ^2_{Bi-S3}	R_{Bi-Bi1}	σ^2_{Bi-Bi1}	R_{Bi-Bi2}	σ^2_{Bi-Bi2}	R_{Bi-Bi3}	σ^2_{Bi-Bi3}
Bi_2S_3	2.643 (16)	0.009 (1)	3.037 (26)	0.020 (3)	3.348 (20)	0.006 (1)	3.950 (27)	0.005 (4)	4.122 (83)	0.004 (7)	4.356 (78)	0.006 (7)

Sample	R-factor	R_{Bi-S1}	σ^2_{Bi-S1}	R_{Bi-S2}	σ^2_{Bi-S2}	R_{Bi-S3}	σ^2_{Bi-S3}	R_{Bi-S4}	σ^2_{Bi-S4}
1	0.018	2.493 (20)	0.008 (1)	2.598 (20)	0.008 (1)	2.681 (20)	0.008 (1)	2.979 (20)	0.008 (1)
2	0.008	2.470 (13)	0.006 (1)	2.575 (13)	0.006 (1)	2.658 (13)	0.006 (1)	2.956 (13)	0.006 (1)
3	0.019	2.519 (18)	0.007 (1)	2.620 (18)	0.007(1)	2.707 (18)	0.007 (1)	3.007 (18)	0.007 (1)
4	0.017	2.455 (32)	0.006 (1)	2.559 (32)	0.006 (1)	2.643 (32)	0.006 (1)	2.940 (32)	0.006 (1)

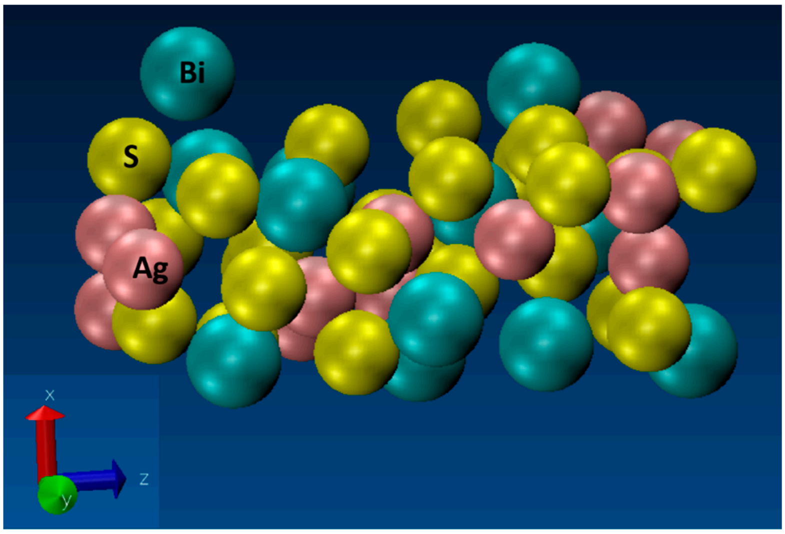

Figure S6. Image of the structure resulting from the MD-DFT simulations.
